# A new test for diagnosing vasovagal syncope: Standing after treadmill test with sublingual nitrate administration

**DOI:** 10.1371/journal.pone.0179631

**Published:** 2017-06-15

**Authors:** Tae-Hoon Kim, Ho-Jun Jang, Sihun Kim, Sung Yun Cho, Kyung Sun Song, Christopher Pickett, Heiko J. Schmitt, Juyong Lee

**Affiliations:** 1 University of Connecticut Health Science Center, Farmington, Connecticut, United States of America; 2 Division of Cardiology, Sejong General Hospital, Bucheon, Gyeonggi-Do, Republic of Korea; 3 Department of Neurosurgery, New Korea Hospital, Gimpo, Gyeonggi-Do, Republic of Korea; Kurume University School of Medicine, JAPAN

## Abstract

**Introduction:**

Increased adrenergic tone might be an additional trigger of orthostatic stress of vasovagal syncope (VVS). Exercise before standing might provide increased sensitivity compared to standing using a sublingual nitroglycerines protocol during tilt table testing. The aim of this study was to evaluate the diagnostic value of treadmill testing before standing with nitroglycerin administration.

**Methods and results:**

A total of 36 patients with syncope or presyncope were enrolled for the test. VVS was confirmed in 29 patients according to the Calgary Score (≥ -2), including 20 patients who were likely to have typical (classical) VVS. All 36 subjects were subjected to a novel provocation test consisting of treadmill test using the Bruce protocol followed by standing with administration of 300 μg sublingual nitroglycerin. Consequently, syncope or presyncope occurred in 22 patients of the 36 patients. The sensitivity and a specificity of the test for Calgary score based VVS was 82.7% and 85.75%, respectively. Reproducibility rate for typical VVS was 90% (18 of 20). In all symptomatic patients, systolic blood pressure dropped to < 90 mmHg and symptom occurred a mean of 6.7 ± 2.3 minutes after the nitroglycerine administration. No patient required anticholinergics injection to restore vital signs.

**Conclusions:**

Treadmill test with administration of sublingual nitroglycerines might be safely used to reproduce syncope in patients with VVS. More clinical experience and confirmation are needed to validate this protocol.

## Introduction

Vasovagal syncope (VVS) is the most common type of reflex syncope. It is usually mediated by orthostatic posture. However, not all patients have syncopal episodes while standing. Increased adrenergic tone such as emotional stress or pain may trigger and contribute to VVS. Although VVS is frequently associated with more complex situations with various stressors and circumstances [1], increased adrenergic tone is thought to be the common trigger toward standing in general. Adrenergic surge after prolonged standing is believed to be a key initiating factor of tilt test using isoproterenol [2]. Currently, tilt test using intravenous isoproterenol or sublingual nitroglycerin is widely used for the patients to confirm VVS. However, the reproducibility rates have stayed at approximately 40~50% for VVS patients in each protocol [3]. We hypothesized that increased adrenergic tone by exercise could be an additional trigger toward the venous pooling stress. If so, the treadmill exercise test (TMT) could be employed to provoke syncope or presyncope in patients suspected to have VVS. The aim of this study was to evaluate the diagnostic value of standing after TMT with sublingual nitrate administration for VVS.

## Materials and methods

### Study population

From August 2015 to July 2016, patients with syncope or presyncope of unknown origin presented to our hospital. Careful history was taken either by the Department of Cardiology or Neurosurgery. Second, physical examination including routine laboratory tests, orthostatic blood pressure (BP) measurements during 3 minutes after standing, electrocardiogram (ECG), and echocardiography were performed for all patients in the Department of Cardiology. Neurologic examination with or without imaging test including brain magnetic resonance imaging and computed tomography were performed in the Department of Neurosurgery. Finally, all subjects without orthostatic hypotension (BP decrease ≥ 20/10 mmHg), organic heart disease, or neurological abnormality were enrolled.

### Study definitions and diagnosis of VVS

Syncope was defined as abrupt loss of consciousness with inability to maintain postural tone but with spontaneous recovery. Presyncope was defined as presence of symptoms of imminent syncope with difficulty in maintaining postural tone [4]. A positive test result of sublingual nitroglycerin TMT was defined as reproducing the spontaneous syncope or impairment of sustain of standing due to symptoms of presyncope regardless of their vital signs. Seven diagnostic questions about medical history, triggers, circumstances, and signs and symptoms of transient loss of consciousness of the Calgary Score were calculated for all patients. Numbers, similarity, and triggers of each event were recorded. VVS was defined as syncope or presyncope if they occurred at least once with a score of ≥ -2 on Calgary syncope symptom scale [5]. Typical (Classical) VVS was defined as transient loss of consciousness triggered by definite causative factors of emotional distress or orthostatic stress [6]. Reflex syncope was diagnosed followed by major causative factors of exercise, gastrointestinal (GI) problems or micturition by history taking. Finally, patients were classified as specific category of syncope according to the guidelines of syncope by European Society of Cardiology (ESC) [7]. If they had more than two episodes of syncope with multiple causative factors, the main or recent causational factor was considered as the major one for classification.

### Provocation test protocol

Patients were given all information about the tests in terms of the purpose of the study, sequence of the test protocol, expected symptoms during and after the exercise test, and the procedure involved. After overnight fasting, intravenous access was obtained for all patients and the procedure was performed in the morning. All drugs were discontinued 3 days prior to the study. All patients signed informed consent before the procedures. The study protocol was approved by the Ethical Committee of Sejong General Hospital. The test consisted of two consecutive phases. After the TMT was performed, standing was the done during the administration of sublingual nitroglycerin. We used the exercise test described in the Bruce protocol. A cardiologist monitored the real time ECG changes at the side of patient to check the presence of arrhythmia or any signs of ischemic heart disease. A goal of over 80% of the max predicted heart rate (220 minus patient’s age) was aimed for each patient. However, it was planned to terminate at the end of the 5th stages (15 minutes of exercise) even if they did not reach the target heart rate.

**Fig 1:**
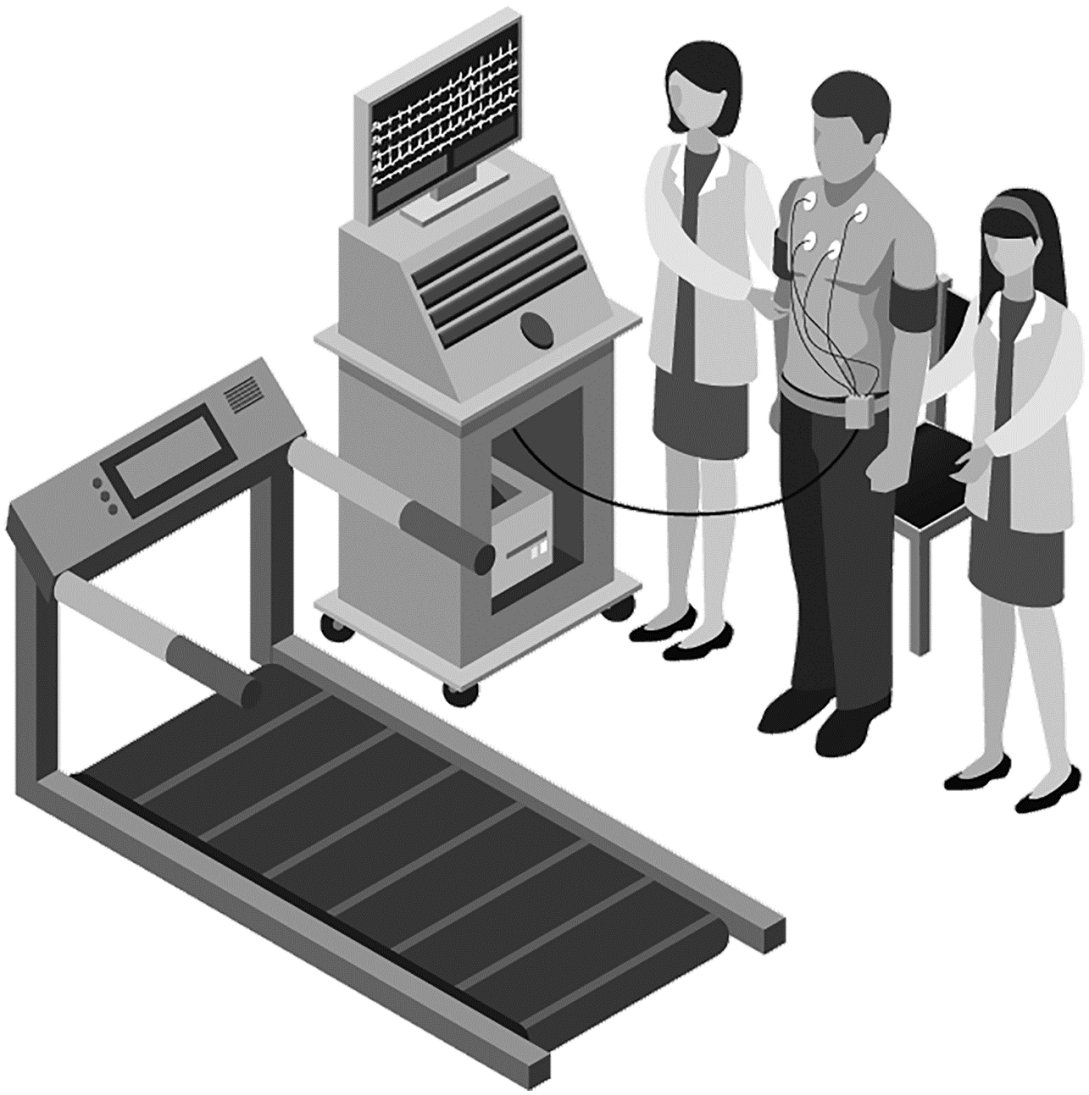
Illustration showing the application of standing test after treadmill test. The patient kept his/her position upright after finishing the treadmill test. Usually, two assistants provided guidance and support. Three hundred micrograms of nitroglycerin was administrated 1 minute after initiating the standing posture. The cuffs for blood pressure measuring (BP) are bilaterally applied either automatically or by the manual measuring method. ECG is continuously recorded during the test.

In the second phase, after finishing their TMT shortly without significant ECG abnormality, patients remained standing on the side of the treadmill machine while keeping their ECG leads on their chest with blood pressure cuffs on both arms (Fig 1). One minute after the exercise while patients where in an upright position, sublingual nitroglycerin 300μg was administrated under continued serial BP measurement and heart rate monitoring. Symptoms were monitored at least every 30 seconds. If patients complained of any discomforts including feeling faint, nausea, and pending syncope, they were asked whether they could continue to stand and how their symptom resembled the previous syncopal episode. Finally, if there were the signs of decrease in systolic BP >50% and decrease in heart rate >30% of the maximal value [4] and/or definite reproductive symptom, the doctor decided to terminated standing. If the doctor decided to terminate standing, final standing vital signs at the time of the symptom were recorded and patients were seated or laid down with continuous monitoring. If they felt faint, near syncopal symptoms, or had syncope, they were positioned supine and their vital signs and consciousness were checked repeatedly. Standing was terminated after 15 minutes if no symptoms occurred. All patients were positioned supine after the test. They were then sent to the ward after symptoms and vital signs stabilized.

### Statistical analysis

Sensitivity and specificity were calculated to evaluate the diagnostic performance of sublingual nitroglycerin TMT based on the results from the Calgary syncope symptom score. To compare characteristics between positive and negative results, continuous data are expressed as mean value ± standard deviation. All categorical data are presented as absolute number and percentage. To evaluate differences between groups, Fisher’s exact test was used for categorical variables while Mann-Whitney U test was used for continuous variables. Significance was considered when *P <* 0.05. Statistical analysis were performed using SPSS 18K.0 (SPSS Inc, Chicago, IL, USA).

## Results

**Fig 2:**
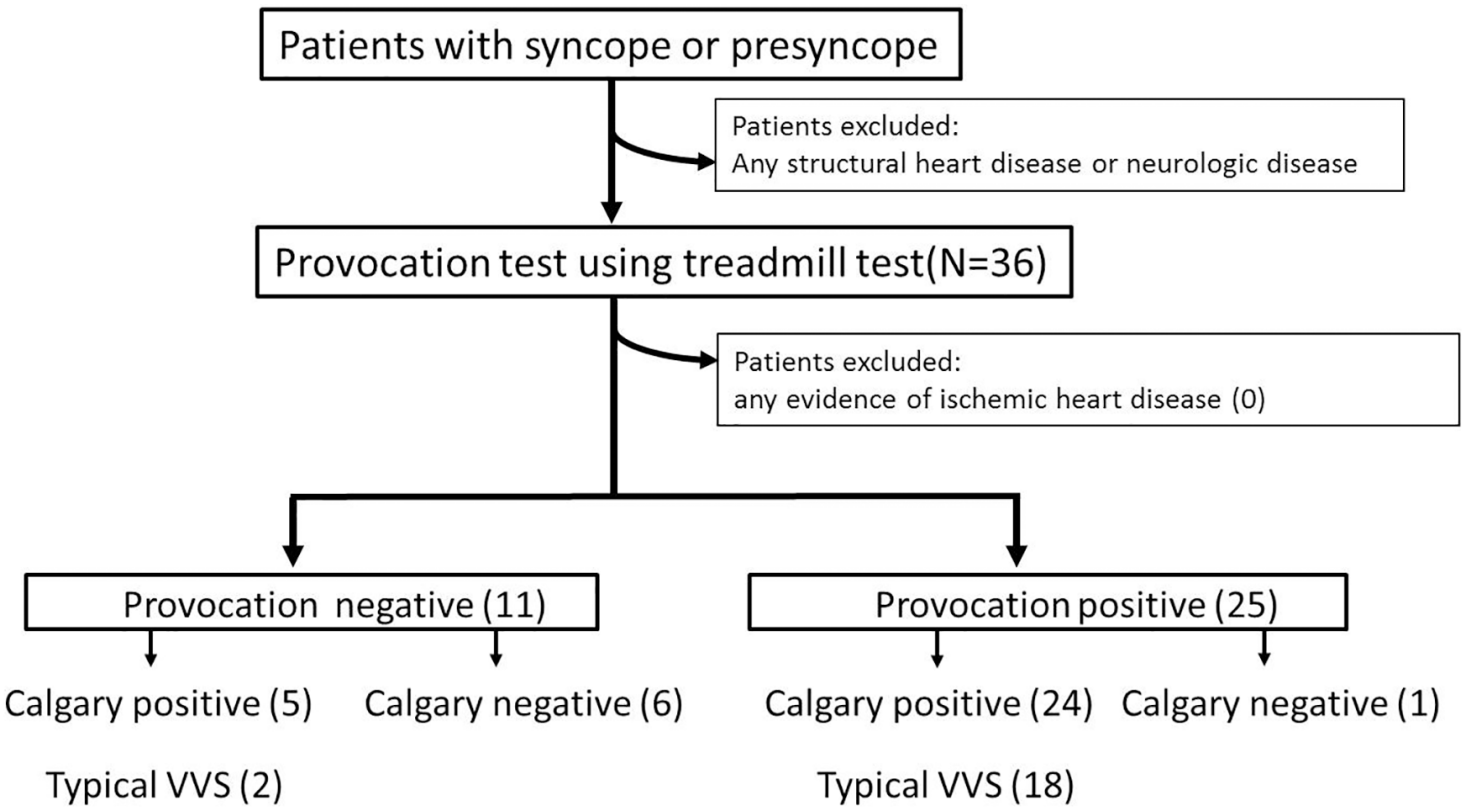
A flow diagram demonstrates the sequence of patients’ enrollment and the test results. The each group’s patients by the provocation test result are classified again by the definition of the Calgary score and that of the typical (classic) vasovagal syncope.

The flow diagram in Fig 2 details the sequence of enrollment and test results compared with the definition of VVS. A total of 36 patients with a history of syncope or presyncope and without any heart diseases and neurologic disorders were enrolled. All 36 patients were eligible for the standing test after TMT and completed the study.

**Fig 3:**
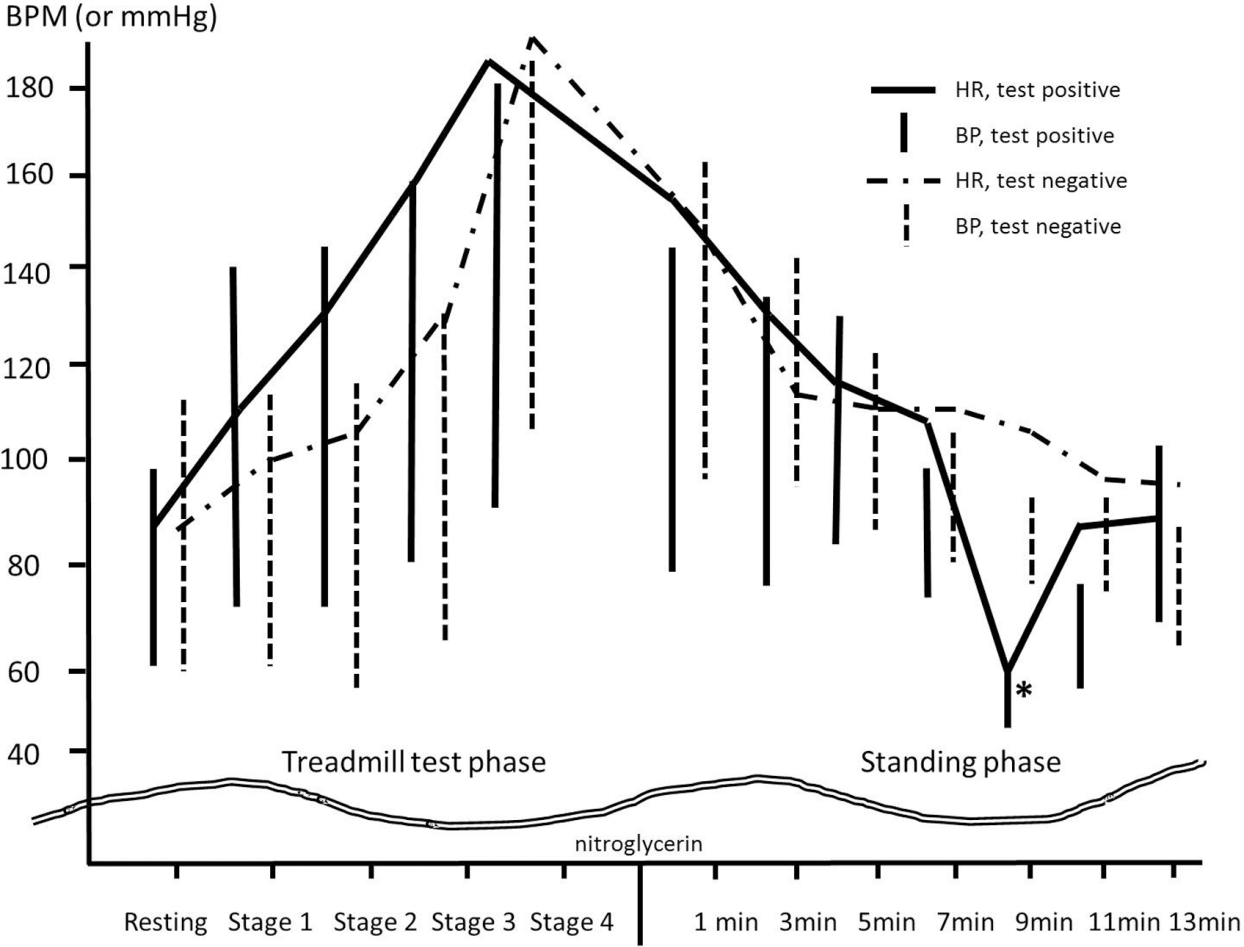
Serial blood pressures (BP) and heart rates (HR) of representative patients in test positive and test negative group. The solid line and the solid bar indicate the HR and BP, respectively, of a 43-year-old male patient with positive result. The dashed line and bar indicate the HR and BP, respectively, of a 33-years-old female patient with negative result. There are gradual decreases of BP and HR after nitroglycerin administration in test negative patient. There are sudden drops of BP and HR at 9 minutes after nitroglycerin administration (asterisk).

The characteristics of the study populations and their diagnosis by history or Calgary scoring system are shown in Table 1. All baseline characteristics were not significantly different. However, age of the provocation negative group tended to be higher. Among the 36 patients, 15 presented a single episode, while 21 patients experienced more than two syncopal or presyncopal episodes. Although 29 patients were diagnosed as VVS by Calgary score system, 9 could be categorized by the reflex syncope from the history of the case and typical VVS was diagnosed in 20 patients among all populations. Reproducibility rate of symptoms through the test reached 90% (18 of 20) of patients in typical VVS. Among the total of 36 patients, syncope or presyncope symptoms were provoked in 25 patients during standing. However, according to the Calgary Score (≥ -2), VVS was identified in 29 patients. Consequently, sensitivity and specificity of the test for Calgary score based VVS were 82.7% and 85.75%, respectively. Although this new diagnostic test also showed a positive predictive value as high as 96.0%, the negative predictive value was 54.55%. Typical (Classical) VVS was diagnosed by the definition in 20 patients in the study population, 18 had a positive result. The remaining 2 did not have symptoms during the study. Therefore, the sensitivity (or reproducibility) of the test was 90% for the typical VVS population. There were no athletes included in the study. However, 7 had more than one event related to exercise or high physical activity. Two were diagnosed as typical VVS. A 44-year-old woman was late for work and ran to catch the subway. She syncopysed as she was standing after boarding the subway car. A 23-year-old man did railroad work in the early morning. He experienced syncope while standing during a break. Both individuals displayed positive test results. Five of the seven were diagnosed as post-exercise syncope by history-taking; only one had a positive test result. All enrolled patients achieved more than 80% of maximum predicted heart rate (HR; average, 99.3%; range, 87% to 110%). Serial BP and HR of representative patients in test positive and test negative group are presented in Fig 3 and results of hemodynamics at baseline, during exercise, and during standing are shown in Table 2. All parameters were not significantly different between groups at baseline. However, maximum HR during exercise in the provocation positive group was higher than that in the provocation negative group. This might be because their age tended to be lower compared to that of the provocation negative group. In the provocation positive group, symptoms occurred at a mean of 6.7 ± 2.3 minutes after the initiation of nitroglycerine administration (minimum 2 minutes, maximum 11 minutes). However, no one developed symptoms during exercise or before nitroglycerin administration. The lowest HR was a mean of 82.4 ± 24.8 beats per minute (range, 30 to 126). One patient in the negative provocation group had short duration of the bigeminy form of premature ventricle contraction during the TMT, but the standing phase with the nitroglycerin safely proceeded without having to stop the test. Although only 3 (12.0%) patients had a systolic pause of > 2 seconds or sinus arrest during the standing phase, all patients in the positive provocation group experienced a drop in systolic BP < 90 mmHg. However, only 45.5% (*p* < 0.001) patients in the negative provocation group had such drop. No adverse events occurred during or after the test. Patients recovered by maintaining their supine positions or by intravenous normal saline administration after the events. No patients required anticholinergics injection to restore their vital signs.

**Table 1:** Characteristics of each group classified by the results of provocation test.

N (%)	Provocation negative (11)	Provocation positive (25)	P value
Age	37.9 ± 13.2	31.0 ± 14.4	0.180
Male	3 (27.3)	10 (40.0)	0.708
Height (cm)	165.2 ± 8.2	166.0 ± 8.5	0.959
Weight (kg)	59.3 ± 10.1	56.1 ± 11.7	0.570
Body mass index	21.6 ± 2.6	20.2 ± 3.4	0.287
Hypertension	0	2 (8)	>0.99
Diabetes	0	0	
Smoking	1 (9.1)	0	0.306
Dyslipidemia	1 (9.1)	1 (4.0)	0.524
Ejection fraction (%)	65.0 ± 5.8	68.6 ± 6.3	0.121
Diagnosis by history			
1. Typical VVS	2 (18.2)	18 (72.0)	0.004
2. Reflex syncope	7 (63.6)	4 (16)	0.008
(A) Post-exercise	4 (36.4)	1 (4)	0.023
(B) Micturition	2 (18.2)	2 (8)	0.570
(C) GI-associated	1 (9.1)	1 (4.0)	0.524
3. Non-diagnostic	2 (18.2)	3 (12.0)	0.631
≥ 2 events	5 (45.4)	16 (64.0)	0.465
Calgary positive	5 (45.4)	24 (96)	0.001
Calgary score	-1.36 ± 2.87	2.4 ± 2.27	<0.001

Categorical data are expressed as numbers (%) and continuous data are expressed as mean ± standard deviation.

Abbreviations: VVS = vasovagal syncope; GI = gastrointestinal; ≥ 2 events = patients having more than 2 events of syncope or presyncope; Calgary positive = Calgary score calculated ≥ -2.

**Table 2:** Hemodynamical results during and after the treadmill test.

Treadmill test	Provocation negative (11)	Provocation positive (25)	P value
Baseline hemodynamics			
Baseline heart rate	83.8 ± 12.8	88.8 ± 14.1	0.481
Baseline systolic BP	111.8 ± 10.0	118.0 ± 18.6	0.363
Baseline diastolic BP	75.0 ± 12.8	71.4 ± 9.4	0.228
During Exercise			
Max predicted HR	182.0 ± 13.2	189.0 ± 14.4	0.175
Max HR	178.3 ± 7.8	187.6 ± 15.8	0.029
% max/max predicted HR	98.1 ± 6.2	95.8 ± 20.9	0.630
Max systolic BP	169.8 ± 18.7	168.2 ± 29.9	0.655
Max diastolic BP	74.0 ± 19.3	76.6 ± 13.9	0.470
Total accomplished Mets	12.5 ± 0.8	12.7 ± 0.9	0.511
Recovery			
Time to symptom occur	-	6.7 ± 2.3	
HR at symptom occur	-	95.1 ± 29.6	
Sinus pause or arrest		3 (12.0)	
Systolic BP at symptom occur	-	71.0 ± 10.8	
Lowest systolic BP	93.0 ± 6.8	71.0 ± 10.8	<0.001
% lowest/baseline systolic BP	83.4 ± 6.1	61.6 ± 13.7	<0.001
n(%), >50% drop from max BP	3 (27.3)	18 (72.0)	0.025
Lowest HR	111.9 ± 14.0	82.4 ± 24.8	0.001
% lowest/baseline HR	134.9 ± 18.0	93.1 ± 26.7	<0.001
Systolic BP drop (<90mmHg)	5 (45.5)	25 (100)	<0.001
Systolic BP drop (<85mmHg)	1 (9.1)	24 (96.0)	<0.001

Categorical data are expressed as numbers (%) and continuous data are expressed as mean ± standard deviation.

Abbreviations. BP = blood pressure; HR = heart rate; Mets = Metabolic equivalents

## Discussion

### Main finding of the study

A test protocol using TMT with sublingual nitroglycerine administration might be a very sensitive tool for reproducing symptoms in patients presenting with VVS, which may be triggered easily by an adrenergic surge. The strengths of this protocol are as follows. Physicians can rule out significant coronary artery disease or exercise-associated serious arrhythmia with a single provocation protocol. Adrenergic surge is achieved by activating the endogenous system instead of intravenous administration of catecholamines with potential side effects. Finally, the duration of the standing test is only 15 minutes. Thus, the test can be added to a routine TMT to significantly shorten the testing time compared to a typical tilt table test

### Current study population based on guidelines

VVS is triggered by emotional distress or standing stress [8]. The term vasovagal is not used for other types of syncope in the same reflex category in current guidelines. However, some patients could be categorized as VVS solely following the Calgary score system, even if their events might be caused by other factors. Importantly, the specificity of the Calgary system only reached 32% in a previous study [9]. Thus, we categorized them by their history and added the definition of typical VVS. Finally, our population had 20 patients with typical VVS, 11 patients with reflex syncope, and 5 non-diagnosed patients. Interestingly, the reproducibility rate was 90% in the typical VVS group and 36% in the reflex syncope group.

### Exercise and syncope

Exertional induced syncope was initially thought to result from hypersensitivity of carotid sinus baroreceptor. However, this reflex mechanism was rejected due to failure of producing syncope in such patients [10]. The concept of a reflex mechanism was revised. In this mechanism, a depressor reflex arising from the stimulation of the left ventricular baroreceptors might be implicated. In a severe aortic stenosis model of animal, increased left ventricular pressure has been demonstrated to be able to promote reflex vasodilatation and hypotension [11]. Exercise induced vasovagal syncope in a normal heart has also been implicated with reactive bradycardia and hypotension considered as key findings. This paradoxical reflex has been reported frequently. The hypotension rate after exercise test has been demonstrated to be as high as 3.1% of all asymptomatic volunteers [12]. In addition, 22% out of 54 syncope patients have their events associated with exercise in a previous study [13].

Tilt table testing is frequently performed to provoke syncope for exercise induced syncope. However, the positive provocation rate is low based on tilt test alone. Other authors required additional isoproterenol infusion for provocation. The current guideline recommends exercise testing for patients who have experienced episodes of syncope during or shortly after exertion [8]. However, this is done mainly to rule out the presence of organic heart disease. Interestingly, in a previous study, reproducing syncope occurred only in one out of 17 exercise-induced syncope patients during exercise testing, although all the patients displayed an abnormal tilt test response [14]. Similarly, although our study protocol utilized nitroglycerin administration, symptoms were reproduced during testing in only one of 5 post-exercise syncope patients.

### Tilt test protocol for VVS

Provocative tests aim to reproduce syncope or presycope in a laboratory setting. The assumption is that a positive response to a test can reproduce the mechanism of a spontaneous episode. The tilt test was introduced by Kenny et al. in 1986 [15]. It has become an essential diagnostic tool to reproduce syncope in VVS. It could be applied to other forms of reflex syncope [16]. However, usually low-dose intravenous isoproterenol [2] or 300~400 μg of sublingual nitroglycerine [17] is required to increase positive response with a high specificity. The basic mechanism of using isoproterenol is that VVS episodes are preceded by an increase of adrenergic tone by tachycardia. However, adverse side effects of intravenous injection of isoproterenol have been reported up to 16% of patients [18]. Therefore, exclusion of significant coronary artery disease is preferable. Moreover, the specificity of the test for relatively younger patents has been questioned [19]. The nitroglycerine-based protocol is based on the increase of venous pooling stress in addition to orthostatic stress. Sublingual nitroglycerin is more easily and safely applied and is better tolerated than isoproterenol. It leads to a higher number of positive responses than isoproterenol in comparison studies [3,20]. However, its positive provocation rate was in the range of 51~55% [4]. To address the low reproduction rate, many authors have been tried different protocols [21], however the reproducibility of the test was not significantly changed [22]. It is thought to have higher positive provocation rate if venous pooling stress is enhanced by the nitroglycerin added to adrenergic stimulation. Consequently, our protocol safely utilized TMT and has increased a reproducibility rate of 82.7% for Calgary score based VVS and 90% for patients with typical VVS.

### Current protocol in terms of pathophysiology of VVS

Acute central hypovolemia and vigorous myocardial contraction are believed to be key initiating factors of VVS. Although they similarly contribute to the development of VVS, provocation test using isoproterenol always utilizes a venous pooling phase preferential to vigorous contraction. In contrast, the basic concept of our protocol was that we first utilized sympathetic activation for venous pooling using a TMT. TMT is one of the best diagnostic tools for evaluating the presence of coronary artery disease or arrhythmia. Since our protocol used TMT to provide an adrenergic surge that was more physiological than the previous isoproterenol protocol, it might be less prone to complications with a significantly reduced time needed for diagnosis. Moreover, the presence of coronary artery disease can be evaluated with the same test. Patients with these conditions could be safely excluded before starting the venous pooling phase.

The concept of using TMT with prolonged standing was first introduced by Doi et al. [23]. The approach appeared physiologically acceptable to diagnosis for patients with reflex syncope. Although high specificity (95%) was reported, the exercise test had a markedly lower reproduction rate (sensitivity: 43%). Thus, we utilized sublingual nitroglycerin administration in addition to TMT. Safety of peri-exercise administration of sublingual nitroglycerin has been achieved [24]. Patients’ compliance as well as clinical advantages of this medication was also demonstrated by the exercise test for claudicants and the patients with stable angina [25,26]. Since the define dose of sublingual nitroglycerin for provoking vasovagal syncope has not been studied previously, we tried a similar dosage of sublingual nitroglycerin used in the head-up tilt test by Raviele et al. [4].

Although our study successfully increased the reproducibility of syncopal episode through nitroglycerin with TMT, a low negative predictive value (54.55%) was still evident in the current test protocol. Interestingly, in a previous report [23], the author used an adjusted protocol involving rapid increase of exercise intensity followed by abrupt termination. Only 5 of 44 patients with a history of syncope had the symptom reproduced using the Modified Bruce protocol, while 21 had syncope with the adjusted protocol. The use of our nitroglycerin protocol combined with this exercise protocol might be a solution to patients with a higher threshold.

We also used the Calgary syncope symptom score as the standard for the presence of VVS. The Calgary syncope symptom score was approved by the European Society of Cardiology for use in diagnostic questionnaires for syncope. It has a sensitivity of 89% and a specificity of 91% for VVS after correcting for error [27]. However, specificity of the Calgary score system was lower (32%) when it was applied without confirmation of patients’ history. Thus, we utilized the term typical VVS as a complementary measure.

### Limitation of the study

The number of patients used in this study was small. More information could be provided if this protocol was compared to the tilt test protocol. However, to improve the reliability of the study, we used the Calgary diagnostic score system as well as specific classification as typical VVS.

## Conclusion

TMT with sublingual nitroglycerines administration might be safely used to reproduce syncope in patients with VVS. More clinical experience and confirmation are needed to validate this protocol.

## Supporting information

S1 Dataset De-idintified dataset of the study population. (XLSX)
